# A robust penalized method for the analysis of noisy DNA copy number data

**DOI:** 10.1186/1471-2164-11-517

**Published:** 2010-09-25

**Authors:** Xiaoli Gao, Jian Huang

**Affiliations:** 1 Department of Mathematics and Statistics, Oakland University, Rochester, MI 48309, USA; 2 Department of Statistics and Actuarial Science, University of Iowa, Iowa City, IA 52246, USA; 3 Department of Biostatistics, University of Iowa, Iowa City, IA 52246, USA

## Abstract

**Background:**

Deletions and amplifications of the human genomic DNA copy number are the causes of numerous diseases, such as, various forms of cancer. Therefore, the detection of DNA copy number variations (CNV) is important in understanding the genetic basis of many diseases. Various techniques and platforms have been developed for genome-wide analysis of DNA copy number, such as, array-based comparative genomic hybridization (aCGH) and high-resolution mapping with high-density tiling oligonucleotide arrays. Since complicated biological and experimental processes are often associated with these platforms, data can be potentially contaminated by outliers.

**Results:**

We propose a penalized LAD regression model with the adaptive fused lasso penalty for detecting CNV. This method contains robust properties and incorporates both the spatial dependence and sparsity of CNV into the analysis. Our simulation studies and real data analysis indicate that the proposed method can correctly detect the numbers and locations of the true breakpoints while appropriately controlling the false positives.

**Conclusions:**

The proposed method has three advantages for detecting CNV change points: it contains robustness properties; incorporates both spatial dependence and sparsity; and estimates the true values at each marker accurately.

## Background

Deletions and amplifications of the human genomic DNA copy number are the causes of numerous diseases. They are also related to phenotypic variation in the normal population. Therefore, the detection of DNA copy number variation (CNV) is important in understanding the genetic basis of disease, such as, various types of cancer. Several techniques and platforms have been developed for genome-wide analysis of DNA copy number, including comparative genomic hybridization (CGH), array-based comparative genomic hybridization (aCGH), single nucleotide polymorphism (SNP) arrays and high-resolution mapping using high-density tiling oligonucleotide arrays (HR-CGH) [1-5]. These platforms have been used with microarrays. Each microarray consists of tens of thousands of genomic targets or probes, sometimes referred to as markers, which are spotted or printed on a glass surface. During aCGH analysis, a DNA sample of interest (test sample), and a reference sample are differentially labelled with dyes, typically Cy3 and Cy5, and mixed. The combined sample is then hybridized to the microarray and imaged, which results in the test and reference intensities for all the markers. The goal of the analysis of DNA copy number data is to partition the whole genome into segments where copy numbers change between contiguous segments, and subsequently to quantify the copy number in each segment. Therefore, identifying the locations of copy number changes is a key step in the analysis of DNA copy number data.

Several methods have been proposed to identify the breakpoints of copy number changes. A genetic local search algorithm was developed to localize the breakpoints along the chromosome [6]. A binary segmentation procedure (CBS) was proposed to look for two breakpoints at a time by considering the segment as a circle [7]. An unsupervised hidden markov model (HMM) approach was used to classify each chromosome into different states representing different copy numbers [8]. A hierarchical clustering algorithm was studied to select interesting clusters by controlling the false discovery rate (FDR) [9]. A wavelets approach for denoising the data was used to uncover the true copy number changes [10]. The performances of these methods were carefully compared [11].

Recently, several penalized regression methods have been proposed for detecting change points. In the framework of penalized regression, a least squares (LS) regression model was used with the least absolute penalty on the differences between the relative copy numbers of the neighboring markers [12]. This model was called the Lasso based (LB) model since it can be recast into LS regression with the Lasso penalty [13]. The LB model imposes some smoothness properties on the relative copy numbers along the chromosome. However, it does not take into account the sparsity in the copy number variations. Here the smoothness means that the nearby markers tend to have the same intensities and there is only a few markers where changes occur; the sparsity means that only a small number of markers have some nonzero intensities. A penalized LS regression with fused lasso penalty (LS-FL) was proposed to detect "hot spot" in a CGH data [14,15]. This method is applied to incorporate both sparsity and smoothness properties of the data. It is well-known that the solutions based on LS framework can be easily distorted by a single outlier. Both LB and LS-FL methods lack robust properties when the data does not have a nice distribution. Considering the possible data contamination in a microarray experiment, quantile regression with Lasso (Quantile LB) method was studied for the noisy array CGH data [16,17]. However, when the data is sparse, the Quantile LB method does not incorporate the sparsity property of the data sets and then tends to identify change points false positively.

In this manuscript, we propose a penalized LAD regression with the adaptive fused lasso penalty to analyze the noisy data sets. We name this method as the LAD-aFL. The proposed LAD-aFL method has three advantages in detecting CNV change points. First, it is expected to be resistant to outliers by using the LAD loss function. Second, the adaptive fused lasso penalty can incorporate both spatial dependence and sparsity properties of CNV data sets into the analysis. Third, the adaptive procedure is expected to significantly improve the estimates of the true intensity at each marker.

## Methods

### LAD-aFL model for CNV analysis

For a CGH profile array, let *y*_*i *_be the log2 ratio of the intensity of the red over green channels at marker *i *on a chromosome, where the red and green channels measure the intensities of the test (e.g. cancer) and reference (e.g. normal) samples. We assume that those intensities have been properly normalized. Let *β*_*i *_be the true relative copy number and *u*_*i *_(= *β*_*i *_*- β*_*i-*1_) be the true jump value at marker *i *respectively. For the notation's convenience, we denote *β*_0 _= 0 and thus *u*_1 _= *β*_1_. The observed *y_i _*can be considered to be a realization of *β_i _*at marker *i *with a random noise,

(1) $$y_{i}=\beta_{i}+\varepsilon_{i},\;i=1,\cdots n,$$

where *n *is the number of markers on a given chromosome. Our task is to make inference about *β_i_*'s based on the observed *y_i_*'s. There are three possible factors in model (1). First, there may be outliers in the observed data, so a robust procedure is needed. Second, the real *β_i_*'s have the spatial dependence because the true relative copy numbers of the nearby markers are the same except in the regions where the relative copy numbers change abruptly. Third, copy number changes only occur at a few locations in the chromosome; most of the *β_i_*'s should be zero. Based on those three factors, we propose the criterion

(2) $$\sum_{i=1}^{n}|y_{i}-\beta_{i}|+\lambda_{1}\sum_{i=1}^{n}a_{i}|\beta_{i}|+\lambda_{2}\sum_{i=2}^{n}b_{i}|\beta_{i}-\beta_{i}{}_{-1}|.$$

Here, λ_1 _and λ_2 _are two tuning parameters controlling the sparsity and smoothness of the estimates, $a_{i}(=1/|{\hat{\beta }}_{i}^{\left(0\right)}|)$ and $b_{i}(=1/|{\hat{u}}_{i}^{\left(0\right)}|)$ are the weights of two penalties from any consistent initial estimates ${\hat{\beta }}_{i}^{\left(0\right)}$ and ${\hat{u}}_{i}^{\left(0\right)}$. A LAD-aFL estimator of β(=(β_1_, ⋯, β*_n_*)') is the value $\hat{\beta }$ that minimizes (2). In this criterion, we use the absolute loss to reduce the influence of outliers; we use the adaptive fused Lasso penalty, an adaptive version of the fused Lasso penalty, to measure both sparsity and smoothness properties of *β_i_*'s in a CGH data set. By penalizing the term $\sum_{i=1}^{n}a_{i}|\beta_{i}|$ in (2), the sparse solution ${\hat{\beta }}_{i}$'s is expected to have some oracle properties under some conditions [18]. One can understand the oracle properties in the way that the estimates of true nonzero β*_i_*'s in the full model are as well as if the true zero β*_i_*'s are given in advance. If we rewrite (2) as a regression problem of *u_i_*'s, then the term $\sum_{i=2}^{n}b_{i}|\beta_{i}-\beta_{i-1}|(=\sum_{i=2}^{n}b_{i}|u_{i}|)$ provides a measurement of the sparsity of the parameters *u_i_*'s, which reflects the spatial dependence of the true *β_i_*'s. By penalizing this term, the sparse solution ${\hat{u}}_{i}$'s are expected to have some oracle properties under some conditions.

In our study, we set the initial values of ${\hat{\beta }}^{\left(0\right)}$ to be a regular LAD estimator. In other words, ${\hat{\beta }}_{i}^{\left(0\right)}$ = *y_i _*for *i *= 1, 2, ⋯, *n *and ${\hat{u}}_{i}^{\left(0\right)}$ = *y*_*i *_- *y*_*i-*1 _for *i *= 2, ⋯, *n*.

### Computation

Let **y **= (*y*_1_, ⋯, *y*_*n*_)' and a *n *× *n *diagonal matrix $U_{\lambda_{1}}$ = diag (*a*_1_λ_1_/2, *a*_2_λ_1_, ⋯, *a_n_*λ_1_). Define a *n *× *n *matrix $V_{\lambda_{1}}{,}_{\lambda_{2}}$ as

$$\left[\begin{array}{cccccc} \lambda_{1}b_{1}/2 & 0 & 0 & \cdots & 0 & 0\\ -\lambda_{2}b_{1} & \lambda_{2}b_{2} & & \cdots & 0 & 0\\ \vdots & \vdots & \vdots & \ddots & \vdots & \vdots \\ 0 & 0 & 0 & \cdots & -\lambda_{2}b_{n-1} & \lambda_{2}b_{n} \end{array}\right].$$

Consider a new response vector y* = (**y**', **0**', **0**')' and a new design matrix $X^{*}={\left[I,{U^{\prime }}_{\lambda_{1}},{V^{\prime }}_{\lambda_{1},\lambda_{2}}\right]}^{\prime }$, we re-write (2) as

(3) $$L(\beta ,\lambda_{1},\lambda_{2})=|y^{*}-X^{*}\beta |.$$

For every fixed λ_1 _and λ_2_, (3) is the objective function of a LAD regression problem with a new sparse design matrix X*. Therefore, an existing program such as the R quantreg package can be used to compute $\hat{\beta }$.

### Determining the tuning parameters

The magnitude of tuning parameters λ_1 _and λ_2 _determine the smoothness and sparsity of the estimates ${\hat{\beta }}_{i}$'s. In one extreme, if λ_1 _= 0 and λ_2 _= 0, then the estimate of *β*_*i *_is simply *y*_*i*_, which obviously leads to too many estimated non-zero relative ratios. In the other extreme, if λ_1 _is very large, then all ${\hat{\beta }}_{i}$'s are forced to be zero regardless of the data, which is not reasonable.

We provide a fast algorithm to choose tuning parameters in LAD-aFL. For every fixed combo of λ_1 _and λ_2_, we obtain a LAD-aFL solution, ${\hat{\beta }}_{i}$'s, and the complexity of the model, $\hat{df}$. Let $A_{1}=\{1\leq i\leq n;{\hat{\beta }}_{i}=0\}$, $A_{2}=\{1\leq i\leq n;{\hat{\beta }}_{i}={\hat{\beta }}_{i}{}_{-1},\max\{|{\hat{\beta }}_{i}|,|{\hat{\beta }}_{i-1}|\}>0\}$. If we assume that the cardinalities of *A*_1 _and *A*_2 _are *m*_1 _and *m*_2 _separately, then $\hat{df}$ = *n *- *m*_1 _- *m*_2 _[19]. Our analysis shows that the Schwarz information criterion (SIC) works relatively conservative for analyzing the CGH data because of the small number of changes in a data set [20]. We modify SIC as

$$\log\left(\sum_{i=1}^{n}|y_{i}-{\hat{\beta }}_{i}|/n\right)+q*0.5*\hat{df}(log(n)/n),$$

where *q *≥ 1 is a user-defined SIC factor. Larger *q *tends to choose a more parsimonious model. We search the tuning parameters λ_1 _and λ_2 _using the following two steps.

1. Let *q *= *q*_1 _with *q*_1 _≥ 1. For a fixed small value of λ_1_, say λ_1 _= 0.001, we search the "best" λ_2 _from a uniform grid to minimize SIC.

2. Let *q *= *q*_2 _with *q*_2 _≥ 1. For the above "best" λ_2_, we increase λ_1 _by a small increment from a uniform grid and search a "best" one to minimize SIC.

Here λ_2 _controls the frequency of alteration region, and λ_1 _controls the number of nonzero log2 ratios. Noticing that there are much less number of alterations than the number of nonzero log2 ratios in a CGH array data set, we can select λ_2 _more aggressively by choosing *q*_1 _= 1.5 and *q*_2 _= 1 in our computation.

Even though many cancer profiles contain large size of aberrations, which do not have the sparsity in their relative intensities data sets, the existence of the sparsity of the jumps (only a few jumps exists for the relative intensities) still favors the penalized method. To reflect the true relative intensities accurately, we can choose a small λ_1_, say, λ_1 _= 0.001. Our simulations show that LAD-aFL is significantly efficient in mapping these true segments.

### Estimation of FDR

Let ${\hat{\beta }}_{i}$ be the LAD-aFL estimate using the above SIC strategy and ${\hat{\mu }}_{i}(={\hat{\beta }}_{i}-{\hat{\beta }}_{i-1})$ be the estimated jump at marker *i*. The set {1 ≤ *i *≤ *n *: ${\hat{u}}_{i}$ ≠ 0} includes all the potential breakpoints. However, some of the nonzero estimated jumps may not be significant and can lead to false positives. We often treat the question of whether there is a significant copy number change at a position as a hypothesis testing problem [12,15]. The null hypothesis is that the marker *i *does not belong to any gain/loss region. When all the positions are investigated simultaneously, it becomes a multiple testing problem. In this multiple testing problem, FDR is defined as the expectation of the proportion of false positive results, which can be estimated by the number of markers picked under null hypothesis divided by the number of markers picked in the observed data [21-23].

Suppose all nonzero estimates ${\hat{u}}_{i}$'s divide a CGH array into *K *segments, *S*_1_, *S*_2_, ⋯, *S_K _*. The *k*th segment *S_k_*, 1 ≤ *k *≤ *K*, includes *n_k _*markers and has sample median ${\tilde{y}}_{k}$. The hypothesis of interest is

$$H_{0}^{k}:\text{the median of }{\left\{y_{i}\right\}}_{i\in S_{k}}\text{ is equal to }0.$$

We consider the test statistic

z^k=2f^(0)nkβ^˜k,

where β^˜k is the median of all estimated copy number ${\hat{\beta }}_{i}$'s in the *k*th segment and $\hat{f}(0)$ is an estimate of the ordinary of error distribution at 0 in model (1). Using Cox and Hinkley's approach, we have$\hat{f}(0)=(t-s)/[n({\hat{e}}_{\left(t\right)}-{\hat{e}}_{\left(s\right)})]$, where ${\hat{e}}_{\left(i\right)}$'s are ordered sample residuals and *t *and *s *are symmetric about the index of the median sample residuals. Thus ${\hat{z}}_{k}$ is approximated to be a standard normal distribution under $H_{0}^{k}$[24]. A conservative estimator of FDR for a given cutoff value *p *ϵ (0, 1) is,

$$\hat{\text{FDR}}=\frac{n\cdot p}{\sum_{k}n_{k}I(p_{k}\leq p)}$$

where $p_{k}=P(N(0,1)>|{\hat{z}}_{k}|)$. In our study, we choose *p *= 0.002 without other specification.

### Detection the breakpoints

The procedure of detecting breakpoints can be summarized into two steps.

S1. First we use the SIC to compute ${\hat{\beta }}_{i}$'s and ${\hat{u}}_{i}$'s. All markers where both ${\hat{u}}_{i}$ ≠ 0 and ${\hat{\beta }}_{i}$ >*b*_0 _are identified as the candidates of breakpoints, where *b*_0 _is an empirical cutoff threshold for possible amplifications and deletions. Some work suggested that the possible chromosome amplifications and deletions should satisfy log2-ratio> 0.225, which is corresponding to values between 2 and 3 standard deviations from the mean [25]. We choose *b*_0 _= 0.1 conservatively in our experiment.

S2. For the potential breakpoints in S1, we calculate p-values and estimate FDR. The significant breakpoints are identified by controlling FDR.

## Results and Discussion

### Simulation studies

We evaluate the performance of the LAD-aFL method for detecting CNV using three simulation examples. In the first two examples, we consider 500 markers equally spaced along a chromosome.

All observed log2 ratios are generated from

(4) $$y_{i}=\beta_{0i}+\varepsilon_{i},\;i=1,\cdots ,500.$$

where *β*_0*i*_'s are the true log2 ratios of all 500 markers which have three altered regions corresponding to quadraploid, triploid and monoploid states. Similar to [12], we generate random noises *ε_i_*'s from AR(2), AR(1) and independent models, respectively.

**Example 1**. *To demonstrate the performance of the LAD-aFL method under both sparsity and smoothness conditions, we set the true log2 ratios β*_0*i*_*'s in (4) to be significantly sparse as in Table *1*. We generate ε_i_'s from the following three models such that they have the same standard deviations*.

**Table 1:** The true log2 ratios for Examples 1 and 2

*i*	1-100	101-110	111-450	451-460	461-980	981-1000
*β*_0*i*_	0	1	0	0.59	0	-1

*Independent: ε*_*i *_= *e*_*i*0_,

*AR *(*1*): *ε_i _*= 0.60*ε*_*i-*1 _+ *e*_*i*1_,

*AR *(*2*): *ε_i _*= 0.60*ε*_*i-*1 _+ 0.20*ε*_*i-*2 _+ *e*_*i*2_,

*where e*_*i*0 _~ *N*(0, 0.065^2^), *e*_*i*1 _~ *N*(0, 0.082^2^), *and e*_*i*2 _~ *N*(0,0.1^2^) *for i *= 1, ⋯, 500.

**Example 2**. *In this example, we use the same β*_0*i*_*'s as in Example 1. However, to evaluate the robust-ness property of the LAD-aFL estimator, we simulate e_ij_'s from double exponential (DE) distributions such that ε_i_'s have equal standard deviation *0.1.

*Independent: ε_i _*= *e*_*i*0_,

*AR *(*1*): *ε_i _*= 0.60*ε*_*i-*1 _+ *e*_*i*1,_

*AR *(*2*): *ε*_*i *_= 0.60*ε*_*i-*1 _+ .20*ε*_*i-*2 _+ *e*_*i*2_,

where *e*_*i*0 _~ *DE*(0, 0.0707), *e*_*i*1 _~ *DE*(0, 0.0566) *and e*_*i*2 _~ *DE*(0, 0.0460) for *i *= 1, ⋯, 500.

**Figure 1:**
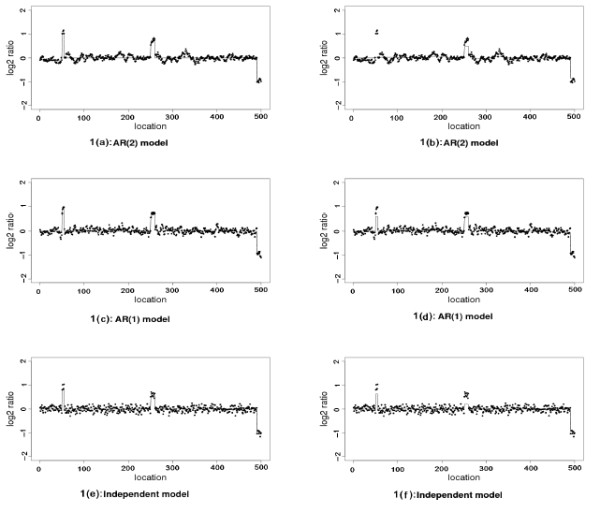
**Analysis for simulated data in Example 2**. 1(a)-(b) AR(2) model. 1(c)-(d) AR(1) model. 1(e)-(f) Independent model. The left and right panels are the results from LAD-aFL and LS-FL respectively. Black dots are the observed log 2 ratios. The estimates from each method are connected by solid lines.

We generate 40 data sets for each model defined in Examples 1 and 2. Our simulated data sets are sparse with two amplifications and one deletion, and only 5 true breakpoints for each data set. Both LAD-aFL and LS-FL method are applied to all three models. In Figure 1, we plot a sample data from Example 2 with both the LAD-aFL and LS-FL estimates. The simulation results are summarized in Table 2. For each model, we calculate the average number and standard deviation of all detected breakpoints from 40 data sets. The average number of correctly and falsely detected breakpoints are also reported.

**Table 2:** Simulation results for Examples 1 and 2

	Methods	AR(2)	AR(1)	**Ind**.
Example 1	LAD-aFL	5.225 (0.831)^1^	5.375 (0.806)	4.750 (0.669)
		4.925^2^, 0.300^3^	4.975, 0.400	4.750, 0
	LS-FL	4.250 (1.149)	4.750 (0.707)	4.550 (0.959)
		4.250, 0	4.725, 0.025	4.525, 0.025
	LAD-FL	5.025 (0.479)	4.975 (0.806)	4.350 (1.167)
		4.850, 0.175	4.900, 0.075	4.275, 0.075

Example 2	LAD-aFL	5.275 (0.598)	5.475 (0.784)	4.925 (0.350)
		5.000, 0.275	4.925, 0.550	4.900, 0.025
	LS-FL	2.850 (1.189)	3.750 (1.171)	3.125 (1.362)
		2.850, 0	3.750, 0	3.125, 0
	LAD-FL	4.850 (0.533)	4.800 (0.791)	4.575 (0.874)
		4.850, 0	4.575, 0.225	4.450, 0.125

^1^The average number (with standard deviation) of all detected breakpoints;

^2 ^The correctly detected breakpoints of the true breakpoints on average;

^3^The falsely detected breakpoints on average.

Our simulation results show that the LAD-aFL method can detect the copy number variations with significant accuracy. Compared to the LS-FL method, LAD-aFL is more stable and robust, even if the simulated data is generated from an independent model. The LS-FL method tends to over-smooth the data set and does not have the robust property. To contain some robust properties, the Loess technique was imposed [15]. Our simulation results show that the LS-FL method with the Loess technique is unstable and may miss many significant breakpoints when the data is significantly sparse. For example, for AR(2) model in Example 2, out of 5 true breakpoints, LAD-aFL detect 5.275 breakpoints on average with standard deviation 0.598, while LS-FL only detect 2.850 breakpoints on average with standard deviation 1.189.

In Table 2, we also provide the simulation results from the LAD-FL method. The LAD-FL method is comparable to the LS-FL with Loess in Example 1 and competent to the LS-FL with Loess in Example 2; it can be explained by the natural robust property of the LAD part. Furthermore, due to the adaptive procedure, the LAD-aFL is more accurate than the LAD-FL in detecting the significant breakpoints in both examples.

In the following Example 3, we apply LAD-aFL to large size aberrations with 10,000 markers equally spaced along a chromosome.

Example 3. *We simulate e_ij_'s from AR(1) model in Example 2. We consider three cases of large aberrations containing 99.8%, 80% and 50% of the probes, respectively, in each profile*.

$$\begin{array}{l} Case\;I:\underset{10}{\underset{︸}{0,\cdots ,0,}}\underset{9980}{\underset{︸}{-0.59,\cdots ,-0.59,}}\underset{10}{\underset{︸}{0,\cdots ,0}}\\ Case\;II:\underset{2000}{\underset{︸}{0,\cdots ,0,}}\underset{8000}{\underset{︸}{-0.59,\cdots ,-0.59.}}\\ Case\;III:\underset{5000}{\underset{︸}{0,\cdots ,0,}}\underset{5000}{\underset{︸}{0.59,\cdots ,0.59.}} \end{array}$$

**Figure 2:**
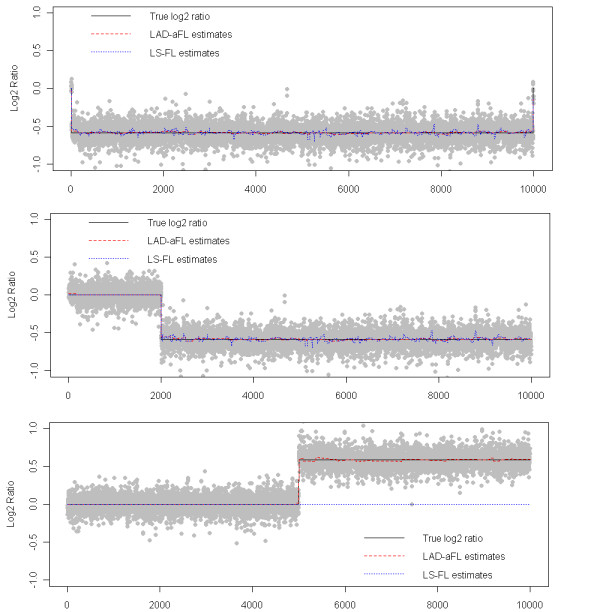
**Analysis for simulated data in Example 3**. The top, middle and bottom panels are for case 1, 2 and 3, respectively. Gray dots are the observed log2 ratios. Black, red, and blue lines represent the true signal, estimates from LAD-aFL, and estimates from LS-FL, respectively.

We summarize the simulation results in Table 3. In all three cases, LAD-aFL can detect the breakpoints accurately. Furthermore, LAD-aFL significantly improves the estimation of the relative intensities for all large aberrations. The sample estimation results of three data sets, with one in each case, are plotted in Figure 2. It is observed that LAD-aFL reflects the true segments and intensities accurately.

**Table 3:** Simulation results for Examples 3

Methods	Case 1	Case 2	Case 3
True Number^1^	2.000	1.000	1.000
LAD-aFL	1.900 (0.410)^2^	1.000 (0)	1.000 (0)
	1.900^3^, 0^4^	1.000, 0	1.000, 0
LS-FL	1.750 (0.444)	1.150 (0.366)	1.000 (0)
	1.750, 0	0.850, 0.300	1.000, 0

^1^The true true breakpoints number for each data set;

^2^The average number (with standard deviation) of all detected breakpoints;

^3^The correctly detected breakpoints of the true breakpoints on average;

^4^The falsely detected breakpoints on average.

We investigate the estimate of FDR in using above examples. For example, if we control FDR rate at level 0.002, out of 100 iterations of model AR(1) in Example 2 and Case I in Example 3, 90% and 95% of the them have true FDR less than 0.002, respectively.

**Figure 3:**
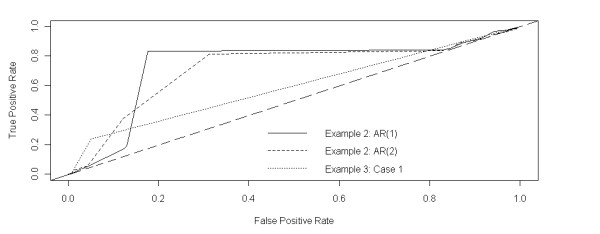
**Roc curve**. The True Positive Rate is computed by the number of probes with true nonzero log2 ratios divided by the total number of probes detected in the aberration region. The False Positive Rate is computed by the number of probes with true zero log2 ratios divided by the total number of probes detected in the aberration region. Three curves are plotted for AR(1) and AR(2) model in Example 2 and Case 1 in Example 3, respectively.

Furthermore, we perform the sensitivity analysis of the LAD-aFL model regarding the cutoff values. In Figure 3, we plot three Receiver Operator Characteristic (ROC) curves for AR(1) and AR(2) models in Example 2 and Case I in Example 3, respectively. We can see that LAD-aFL capture DNA copy number alterations best for AR(1) model in Example 2 and worst for Case I in Example 3.

### Bacterial Artificial Chromosome (BAC) array

The BAC data set consists of single experiments on 15 fibroblast cell lines [25]. Each array contains measurements for 2276 mapped BACs spotted in triplicates. There are either one or two alterations in each cell line as identified by spectral karyotyping with 15 partial and 8 whole chromosomal alterations. The variable used for analysis is the normalized average of the log2 ratio of test sample over reference sample.

**Figure 4:**
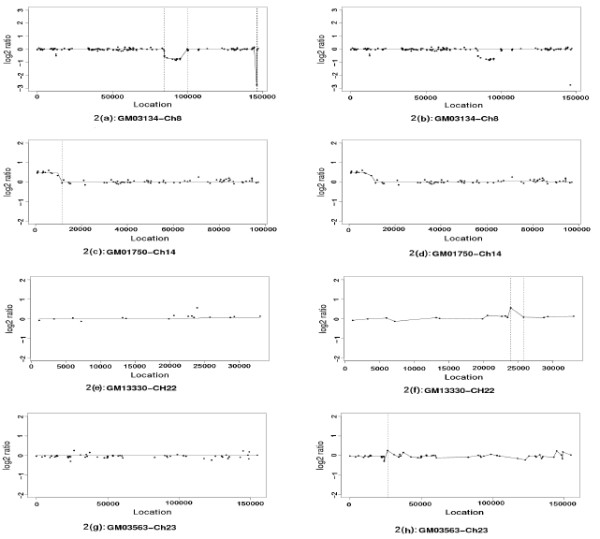
**Analysis of BAC data**. 2(a)-(b) Chromosome 8 of GM03134. 2(c)-(d) Chromosome 14 of GM01750. 2(e)-(f) Chromosome 22 of GM 13330. 2(g)-(h) Chromosome 23 of GM03563. The left and right panels represent results using LAD-aFL and LS-FL methods respectively. Black dots are observed log 2 ratios. The estimates from each method are connected by solid lines. The breakpoints detected by each method are identified by vertical lines.

We applied both LAD-aFL and LS-FL to four chromosomes. Chromosome 8 of GM03134, Chromosome 14 of GM01750, Chromosome 22 of GM13330, and Chromosome 23 of GM03563. Results are demonstrated in Figure 4. Consistent to the Karyotyping method, LAD-aFL detects breakpoints for both Chromosome 14 of GM01750 and Chromosome 8 of GM03134. However, LS-FL tends to over-smooth the estimation around the potential breakpoints and cannot detect any breakpoints. In addition, no breakpoint is detected by LAD-aFL for Chromosome 23 of GM03563 and Chromosome 22 of GM13330, which is also consistent with the result obtained from the Karyotyping method. However, breakpoints are detected by LS-FL for these two chromosomes.

### Colorectal cancer data

**Figure 5:**
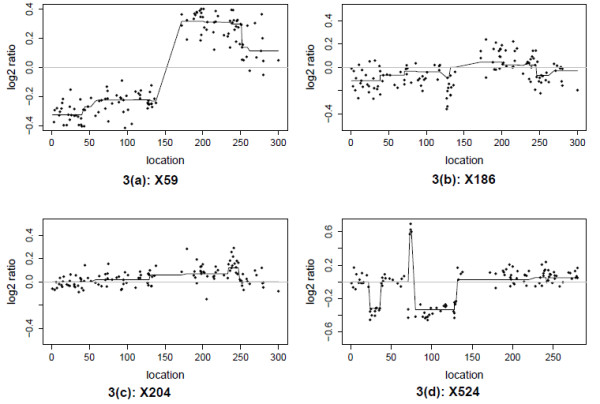
**Colorectal cancer data**. 3(a) X59. 3(b) X186. 3(c) X204. 3(d) X524. Black dots are the observed log 2 ratios. The estimates from the LAD-aFL method are connected by solid lines.

Colorectal cancer data was reported and analyzed for the genomic alterations in tumors of colorectal cancer [16,17,25]. All 125 aCGH DNA data sets are collected using a BAC clone library with clones 1.5 Mb apart and a two-color system with a common reference sample. The available data sets are normalized log2-ratios of sample versus reference per array. There are 133 clones in Chromosome 1. We apply the LAD-aFL to Chromosome 1 in samples X59, X524, X186 and X204. In Figure 5, we plot the estimates of true intensities generated from LAD-aFL. Even though DNA alterations are very common among these aCGH arrays, LAD-aFL can still identify both weak as well as stronger DNA alterations. For example, both X186 and X204 data have unclear pattern, LAD-aFL realizes of the true log2 ratios and reports some weak alterations.

### Human chromosome 22q11 data

High-resolution CGH (HR-CGH) technology was applied to analyze CNVs on chromosome 22q11 [5]. The DNA samples were collected from patients who have Cat-Eye syndrome, 22q11 deletion syndrome (also called velocardiofacial syndrome or DiGeorge syndrome) and some other symptoms. A large proportion of 22q11DS patients develop learning disabilities and attention-deficit hyperactivity disorder with large variations in the symptoms of the disease. For example, patients 03-154 and 97-237 had the typical LCR *A *→ *D *deletion, but they exhibited considerable variation in their symptoms, which might be linked to the deletion size. Therefore, it warrants development of a method which can accurately detect those sizes of deletion regions.

**Figure 6:**
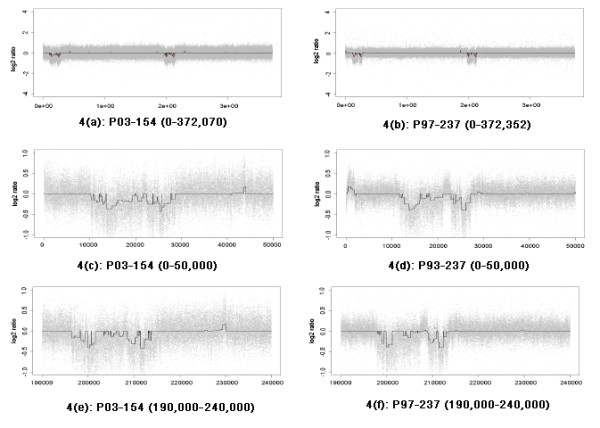
**Human Chromosome 22q11 data sets**. 4(a) Location 0-372,070 of Patient 03-154. 4(b) Location 0-372,352 of Patient 97-237. 4(c) Location 0-50,000 of Patient 03-154. 4(b) Location 0-50,000 of Patient 97-237. 4(e) Location 190,000-240,000 of Patient 03-154. 4(f) Location 190,000-240,000 of Patient 97-237. Here we plot the LAD-aFL analysis of Human Chromosome 22q11 data sets. The left and right panels are results for patient 03-154 and patient 97-237. For each panel, the top, middle and bottom plots show us the results of whole genome, first significant segment (marker 0 - 50,000) and the second significant segment (marker 190, 000 - 240, 000). The observed log 2 ratios are represented by gray dots; the estimates at all markers are connected by solid lines. The cutoff value *p *= 0.0001.

These Human chromosome 22q11 data sets consist of the measurements on chromosome 22 of 12 patients with approximaately 372,000 features in the microarray data sets for each patient. In order to apply the LAD-aFL method, we partitioned the whole chromosome into several segments and then applied the method to each segment. We selected the cutoff value of *p *as 0.0001 since the data set is significantly large and sparse. The LAD-aFL method identified all the blocks previously detected. It also detected the breakpoints for DNA block deletion and amplification. Figure 6 gives the results of the data from patients 03-154 and 97-237. This plot indicates the different deletion sizes in the two patients. In addition, Patient 03-154 appears to have other deleted regions which was not previously detected [5].

## Conclusions

We propose to use a smoothing technique, LAD-aFL to detect the breakpoints, and then divide all the probes into different segments for a noisy CGH data. Very recently, a median smoothing median absolute deviation method (MSMAD) was proposed to improve the performance of breakpoints detection [26]. One can incorporate the LAD-aFL smoother easily into the median absolute deviation process.

The appealing features of the proposed LAD-aFL method include its resistance against outliers, its improved accuracy in mapping the true intensities and the fast and accurate computation algorithm. The robustness property is inherited from LAD regression, which significantly reduces the possibility of false positives due to outlying intensity measurements. These properties are demonstrated in the generating models used in our simulation studies. The adaptive fused Lasso penalty in the LAD-aFL method incorporates both sparsity and smoothness properties of the copy number data. The adaptive procedure generates the solutions with some oracle properties. Computationally, the LAD-aFL estimator can be computed by transform to a unpenalized LAD regression, since both the loss and penalty functions use the same *l*_1 _norm. Our simulation and real data analysis indicate that the LAD-aFL method is a useful and robust approach for CNV analysis. However, there are some important questions which requires further investigation. For example, in the proposed LAD-aFL method, it is assumed that the reported intensity data is properly normalized. It would be useful to examine the sensitivity of the method for different normalization procedures, or perhaps consider the possibility of incorporating normalization into an integrated model. Furthermore, regarding the theoretical properties of LAD-aFL, it would be of interest to consider under what conditions of the smoothness and sparsity of the underlying copy number the LAD-aFL is able to correctly detect the breakpoints with high probability.

## Authors' contributions

XG and JH conceived of the research and designed the study. XG carried out the computational analysis and wrote the paper. JH helped to improve the computational analysis and manuscript preparation. Both authors read and approved the final manuscript.
